# Generating and repairing genetically programmed DNA breaks during immunoglobulin class switch recombination

**DOI:** 10.12688/f1000research.13247.1

**Published:** 2018-04-13

**Authors:** Laura Nicolas, Montserrat Cols, Jee Eun Choi, Jayanta Chaudhuri, Bao Vuong

**Affiliations:** 1 Immunology Program, Memorial Sloan Kettering Cancer Center, New York, NY, USA; 2 Department of Biology, The City College of New York and The Graduate Center of The City University of New York, New York, NY, USA

**Keywords:** immunoglobulin, CSR, AID, DNA repair

## Abstract

Adaptive immune responses require the generation of a diverse repertoire of immunoglobulins (Igs) that can recognize and neutralize a seemingly infinite number of antigens. V(D)J recombination creates the primary Ig repertoire, which subsequently is modified by somatic hypermutation (SHM) and class switch recombination (CSR). SHM promotes Ig affinity maturation whereas CSR alters the effector function of the Ig. Both SHM and CSR require activation-induced cytidine deaminase (AID) to produce dU:dG mismatches in the Ig locus that are transformed into untemplated mutations in variable coding segments during SHM or DNA double-strand breaks (DSBs) in switch regions during CSR. Within the Ig locus, DNA repair pathways are diverted from their canonical role in maintaining genomic integrity to permit AID-directed mutation and deletion of gene coding segments. Recently identified proteins, genes, and regulatory networks have provided new insights into the temporally and spatially coordinated molecular interactions that control the formation and repair of DSBs within the Ig locus. Unravelling the genetic program that allows B cells to selectively alter the Ig coding regions while protecting non-Ig genes from DNA damage advances our understanding of the molecular processes that maintain genomic integrity as well as humoral immunity.

## Introduction

Mammalian adaptive immune responses require B cells to produce immunoglobulins (Igs), commonly known as antibodies, that can recognize a seemingly infinite number of antigens on foreign pathogens. Composed of two heavy (IgH) and two light (IgL) chains that are linked by disulfide bonds, each Ig contains an antigen-binding domain formed from the amino-terminal variable regions of IgH and IgL. The carboxyl-terminal constant (C) region of the IgH chain determines the Ig effector function. Three distinct genomic alterations in the *IgH* and *IgL* loci enable B cells to generate the diverse repertoire of Igs: V(D)J recombination, class switch recombination (CSR), and somatic hypermutation (SHM). During V(D)J recombination, developing B cells in the fetal liver and the adult bone marrow assemble the variable coding regions of IgH from variable (V), diversity (D), and joining (J) coding segments. IgL coding regions are assembled from V and J coding segments in either the *Igκ* or *Igλ* locus. RAG1/RAG2 endonucleases are required for V(D)J recombination, which forms the primary Ig repertoire and promotes the development of mature IgM/IgD-expressing B cells ^1, 2^. Mature B cells with membrane-bound IgM or IgD (B-cell receptor [BCR]) (or both) will migrate to secondary lymphoid organs, such as the spleen, lymph nodes, and Peyer’s patches, where binding of the IgM or IgD to its cognate antigen in the presence of helper T cells will promote CSR and SHM.

CSR reorganizes the *IgH* gene locus to delete the default Cμ/Cδ constant coding exons for an alternative set of downstream constant coding exons (Cγ, Cε, or Cα) ^3^. The B cell thus will switch from expressing IgM or IgD to IgG, IgE, or IgA. Each Ig isotype regulates different effector functions that are necessary for an effective adaptive immune response ^4^. At the molecular level, CSR is a deletional-recombination reaction that occurs at repetitive DNA regions called switch (S) regions, which precede each constant coding exon except Cδ. The intronic region preceding Cδ is a non-canonical, S-like sequence known as σ _δ_. The expression of Cδ, and consequently IgD, is primarily independent of CSR and results from alternative splicing of a primary transcript that includes Cμ and Cδ; however, recent work has shown that CSR to IgD is a rare event confined to mucosa-associated lymphoid tissues and depends on p53 binding protein 1 (53BP1) and myeloid differentiation primary response gene 88 (MyD88) ^5^.

**Figure 1.**
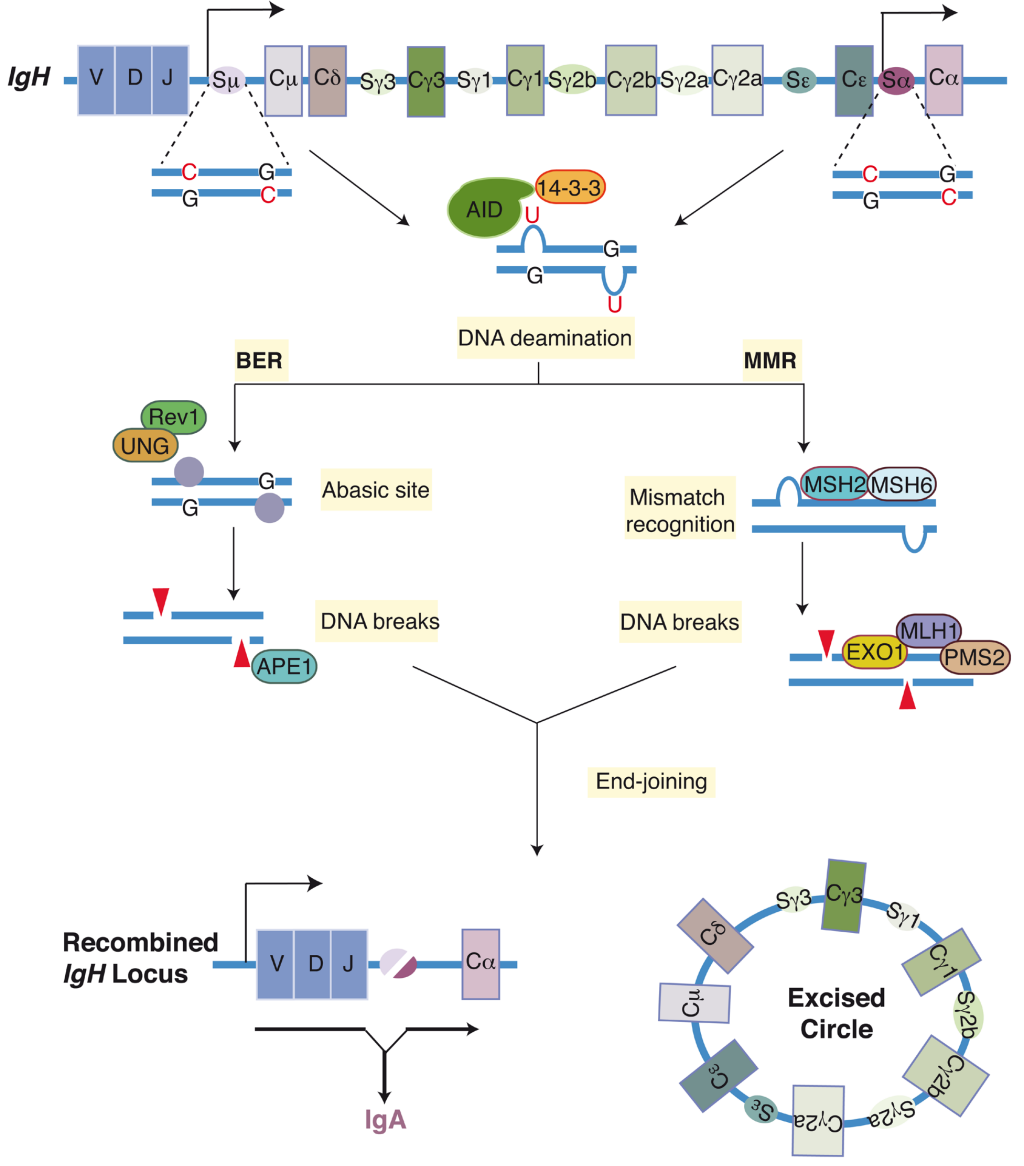
Mature B lymphocytes undergo class switch recombination (CSR) to alter the expression of the immunoglobulin heavy chain constant region (C _H_). The figure depicts CSR between Sμ and Sα in the immunoglobulin heavy chain ( *IgH*) locus. Activation-induced cytidine deaminase (AID) converts cytidines into uridines in S-region DNA. The dU:dG mismatch is converted into DNA double-strand breaks by either the base excision repair (BER) or the mismatch repair (MMR) pathway. In the BER pathway, uracil DNA glycosylase (UNG) removes the uracil base from the DNA to generate an abasic site, which is recognized and cleaved by the apurinic/apyrimidinic endonuclease 1 (APE1). During MMR, the dU:dG mismatch is recognized by mutS homologue 2 and mutS homologue 6 (MSH2 and MSH6), which recruit the complex of exonuclease 1 (EXO1), mutL homologue 1 (MLH1), and post-mitotic segregation 2 (PMS2) to excise a short patch of DNA that includes the dU:dG mismatch. The DNA breaks are ligated by classical or alternative non-homologous end-joining pathways to generate a recombined *Igh* locus and an excision circle. Rev1 and 14-3-3 are scaffolding proteins, which are necessary for the assembly of the protein complexes participating in CSR.

To initiate CSR, DNA double-strand breaks (DSBs) are generated in an upstream donor S region (for example, Sμ) and a downstream acceptor S region (for example, Sα) ( Figure 1). The DSBs are ligated by proteins of the classical-non-homologous end-joining (C-NHEJ) and alternative-NHEJ (A-EJ) pathways, and the sequence between the recombining S regions is excised as an extrachromosomal, circular DNA, which is lost during cell division and DNA replication. Unlike CSR, SHM introduces untemplated point mutations, and occasional deletions and insertions, into the recombined V, D, and J coding exons of *IgH* and *IgL* genes at a very high rate (10 ^−2^–10 ^−3^ base pairs per generation) ^3, 6^. These mutations, which occur primarily in complementarity-determining regions, allow the generation of Igs with an increased affinity toward their cognate antigen.

Both CSR and SHM require activation-induced cytidine deaminase (AID), a 24-kDa protein expressed primarily in activated B cells ^7, 8^. AID, a single-stranded DNA (ssDNA) cytidine deaminase, initiates CSR and SHM by converting deoxycytidine (dC) to deoxyuridine (dU) in recombining S regions during CSR or recombined V(D)J coding exons during SHM. The AID-generated dU:dG mismatch activates DNA repair pathways, including the base excision repair (BER) and mismatch repair (MMR) pathways, which induce DSBs to drive CSR ( Figure 1) or error-prone repair to promote SHM ^9^.

This review describes the general mechanisms of CSR and highlights recent data on the localization of AID to S regions and the DNA repair pathways that resolve AID-generated dU:dG lesions. For an overview of SHM, readers are referred to other reviews ^3, 4^.

## AID targeting to switch regions

Although S regions and Ig variable coding segments are physiological targets of AID during CSR and SHM, respectively, AID can generate DSBs and mutations in non-Ig genes, such as *Myc* and *Bcl6* ^10– 13^. Despite the markedly lower rate of DSB formation and mutation at these non-Ig genes ^13, 14^, the resulting DNA translocations or mutations in these off-target genes contribute to the development of mature B cell lymphomas ^15– 17^. Thus, mechanisms target AID specifically to the Ig loci to promote CSR and SHM while restricting AID access to the remainder of the B cell genome to limit off-target DSBs and mutations to maintain genome integrity.

### Role of germline transcription in generating AID substrates

Ig heavy chain constant (C _H_) exons are organized as independent transcriptional units composed of a cytokine-inducible promoter upstream of a non-coding “I-exon”, the intronic S region, and the corresponding C _H_ exons ^18^. T cell–dependent (for example, cytokines and CD40L) or T cell–independent (for example, lipopolysaccharide) stimuli (or both) activate transcription of recombining S regions ( Figure 1), which is absolutely required for CSR. The primary germline transcript is spliced into a mature, polyadenylated transcript with no known protein product and is frequently referred to as a “sterile” germline transcript ^19^. Genetic deletion of specific I-exons abolishes germline transcription and CSR to the corresponding isotype ^20, 21^. Germline transcription initiating from the I-exons and proceeding through the S regions to the C _H_ exons creates the ssDNA substrates for AID within the transcribed S regions. Each S region varies in length (1–10 kb) and consists of tandem repetitive units that contain a G-rich non-template strand. Deleting the repetitive units within the S regions or replacing the S regions with small core S-region sequences significantly impairs CSR and demonstrates an essential role for these sequences during CSR ^22– 25^. Recent data suggest that the repetitive, G-rich non-template strand forms G-quadruplex (G4) structures that facilitate cooperative AID oligomerization at S regions ^26^. In addition, the tandem repeats of 5′-AGCT-3′ within the core S regions recruit AID and its kinase, protein kinase A (PKA), to the S regions via the 14-3-3 adaptor proteins, which specifically recognize the 5′-AGCT-3′ repeats (Xu *et al.*, 2010 ^27^; reviewed in Xu *et al.*, 2012 ^28^).

Germline transcription of S regions creates R-loops, wherein the newly transcribed RNA hybridizes to the template DNA to form a stable RNA:DNA hybrid that exposes the non-template DNA as ssDNA, which is the substrate for AID ^24, 29– 32^. Inversion of the mouse Sγ1 sequence, which converts the G-rich non-template strand to a G-rich template strand, impairs R-loop formation and CSR without affecting germline transcription ^24^. These data demonstrate the inherent ability of G-rich S regions to form R-loops that likely contain G4 structures, which facilitate AID recruitment ^26, 33^.

Although R-loop formation at non-Ig loci may redirect AID activity to other regions of the B cell genome ^34^, AID is significantly enriched at the *Igh* locus, suggesting that factors beyond R-loop formation also restrict AID to the *Igh* locus during CSR ^14, 35^. AID interacts with RNA polymerase II and its associated proteins, such as Spt5 ^36^, PAF1 ^37^, and the FACT histone chaperone complex ^38^. In addition, RNA polymerase II, which has stalled at the repetitive G/C-rich S regions, can recruit the RNA exosome to degrade the nascent RNA transcript and facilitate AID deamination of the non-template and template DNA strands ^39, 40^.

### Role for germline transcripts in targeting AID to S regions

Germline transcription is necessary but not sufficient for CSR. In mice that lack the Iγ1 exon splice donor site, CSR to IgG1 was abolished despite active S-region transcription ^41– 43^, suggesting that either the RNA processing machinery (for example, spliceosome) or the processed transcripts are required for CSR. CTNNBL1, a component of the spliceosome, interacts with AID and is required for CSR and SHM ^44^. Knockdown of the splicing regulator PTBP2 reduces AID at S regions and impairs CSR ^45, 46^. These data demonstrate that the spliceosome plays an essential role in localizing AID to S regions.

**Figure 2.**
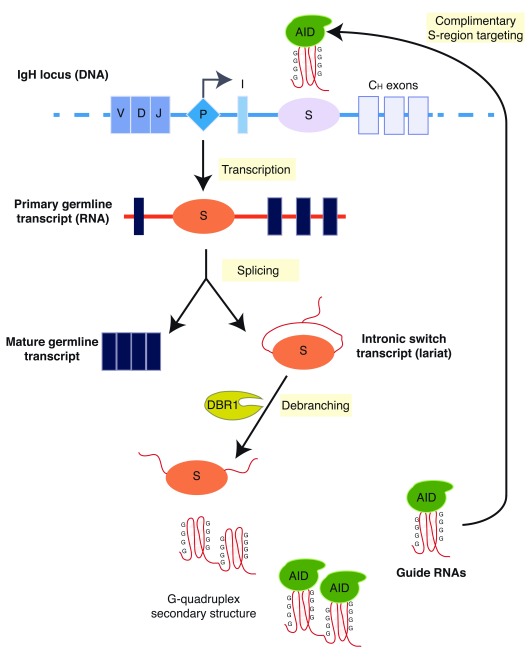
Proposed model for RNA-dependent targeting of AID during class switch recombination. Upon B-cell activation, germline transcription is initiated from a cytokine-inducible promoter (P) and primary germline transcripts are generated from the I-S-C _H_ sequences, which encode the I-exon, switch (S) region, and constant coding exons (C _H_). These transcripts are spliced to form a mature non-coding, germline transcript and an intronic S lariat. The latter is further processed by the debranching enzyme DBR1 to form a linear S-region transcript. Linear S transcripts fold into G-quadruplex RNA, which is bound by AID. The complex of S-RNA and AID is guided to transcribed S-region DNA as a result of the complimentary between the S-RNA and the transcribed S region. AID, activation-induced cytidine daminase; DBR1, debranching RNA lariats 1.

Sα RNA expression from a plasmid in *trans* enhanced CSR to IgA in the B-cell line Bcl _1_B _1_^47^, suggesting that spliced, intronic S-region RNA derived from germline transcripts have a functional role during CSR. More recently, these S-region RNAs were shown to recruit AID to S-region DNA sequences ^33, 48^. Intronic switch RNAs were known to be spliced from primary transcripts to form lariats that undergo hydrolytic degradation, which is catalyzed by the debranching enzyme DBR1 ^49^. Knockdown of DBR1 in CH12F3 cells reduces CSR; however, expression of switch RNAs in *trans* bypasses the lariat debranching step in DBR1 knockdown cells to rescue both CSR and AID recruitment to S regions in a sequence-specific manner ^33, 50^. In addition, AID bound directly and selectively to sense S-region transcripts, which were shown to form highly stable four-stranded G4 structures ^33, 50^. A putative G4 RNA binding motif in AID was identified and mutations in this domain abrogated AID interactions with G4 switch RNA and consequently the localization of AID to S regions and wild-type levels of CSR ^33, 50^. Interestingly, a mutation in the RNA binding motif of AID (G133V) has been identified in patients with Hyper-IgM Syndrome who show severe CSR defects ^51^. From these studies, a new regulatory model for AID localization to S regions was proposed whereby the non-coding, intronic S-region RNA, which is produced following germline transcription and splicing, binds to AID to target AID to sites of DNA recombination (S regions) and promote AID-mediated DNA deamination and CSR in a sequence-specific manner. This model connects the data demonstrating the role of germline transcription and splicing in CSR with the binding of AID to S-region RNA and DNA and identifies a critical function for S-region RNA in CSR beyond germline transcription and splicing ( Figure 2).

### Epigenetic regulation of AID localization to S regions

Epigenetic modifications of the *Igh* locus during CSR have been proposed to control the recruitment of AID to S regions (comprehensively reviewed in 52). Changes in histones H4 and H3 methylation patterns have been associated with altered levels of CSR, although the functional significance of these modifications during CSR remains unclear. Conditional deletion of two methyltransferases ( *Suv4-20h1* and *Suv4-20h2*), which are responsible for histone H4 lysine 20 di- and tri-methylation (H4K20me2 and H4K20me3), in B cells leads to a 50% reduction in CSR ^53^. AID interacts with SUV4-20H1 and SUV4-20H2 in 293F cells and localizes these methyltransferases to S regions to promote SUV4-20-mediated histone trimethylation in B cells undergoing CSR ^54^, suggesting cooperative targeting of methyltransferases and AID to recombining S regions. H3K9me3 tethers AID to Sμ through its interaction with KRAB domain-associated protein 1 (KAP1) and heterochromatin protein 1 (HP1) ^55^, and combinatorial phospho-Ser10 and acetyl-Lys9 modification of H3 (H3K9acS10ph) mediates AID recruitment to S regions by stabilizing S-region DNA binding of 14-3-3, which in turn interacts with AID ^56^. Enrichment of H3K9me3, histone H3 lysine 9 acetylation (H3K9ac), and histone H3 lysine 4 trimethylation (H3K4me3) at recombining, transcribed S regions ^57– 59^ and a reduction in CSR in B cells deficient in Pax interaction with transcription-activation domain protein-1 (PTIP), which is responsible for H3K4 methylation, suggest additional epigenetic mechanisms of regulating AID localization to S regions ^57^. However, the functional relevance of H4K20 and H3K9 methylation in the recruitment of AID to S regions during CSR remains unclear, as some data demonstrate that H3K9 tri-methylation and H4K20 methylation (mono-, di-, and tri-methylation) are reduced at recombining S regions ^55, 60^. Additional work is required to decipher the epigenetic code at S regions, which will further elucidate the role of post-translational modification of histones in the localization and stabilization of AID at S regions during CSR.

## Multiple DNA repair pathways in CSR

Although AID localization to and deamination of S-region DNA is required for CSR, additional factors downstream of germline transcription and AID recruitment are necessary for wild-type levels of CSR ^3, 45, 61, 62^. The conversion of deaminated DNA into DSBs requires many proteins from DNA repair pathways that have evolved to respond to general DNA damage. The mechanism by which these factors convert deaminated DNA into recombinogenic DNA repair (that is, CSR) rather than canonical DNA repair (that is, restoration of the dC:dG base pair at the site of deamination) remains unknown. Below, we discuss our current knowledge of the DNA repair pathways that are required for CSR and highlight the role that AID phosphorylation plays in the generation of DSBs during CSR.

### Converting deaminated DNA into DSBs by BER and MMR

CSR requires BER and MMR pathways to generate DNA breaks in recombining S regions. Defects in either BER or MMR alone significantly impair CSR, whereas combined BER and MMR deficiency (for example, *UNG ^−/−^MSH2 ^−/−^*) completely blocks CSR *in vitro* and *in vivo* ^9, 63^. In the BER pathway for CSR, AID-generated dU in S-region DNA is removed by uracil DNA glycosylase (UNG) to generate an abasic site, which is cleaved by the apurinic/apyrimidinic endonuclease (APE1) to create a single-strand break (SSB) in the DNA ^9, 64– 66^ ( Figure 1). Adjacent SSBs on complementary DNA strands constitute a DSB, which is an obligate intermediate in CSR. Human and mouse B cells with inactivating mutations in UNG exhibit impaired DSB formation at S regions and a severe block in CSR ^66– 69^. Impaired recruitment of UNG to recombining S regions in Rev1-deficient B cells reduces CSR *in vitro* and *in vivo* ^70^. Likewise, mice heterozygous for an APE1 null mutation and CH12F3 cells with a homozygous deletion of APE1 have significantly diminished CSR ^71– 73^.

The AID-generated dU:dG mismatch can also be processed into SSBs through MMR ^9^. In this pathway, an MSH2-MSH6 heterodimer recognizes the dU:dG mismatch and recruits a complex of MLH1/PMS2/EXO1 to repair the mismatch ( Figure 1). PMS2 (PMS1 homolog 2) generates a SSB distal to the mismatch and subsequently exonuclease 1 (EXO1) converts the DNA breaks into ssDNA gaps by excising the segment of the DNA containing the dU in a 5′-to-3′ direction ^3^. EXO1 excision of dU-containing sequences on opposite DNA strands thus would generate DSBs that are required for CSR ^18, 74^. Consistent with this proposed role for PMS2 and EXO1 in converting deaminated S regions into DSBs, humans or mice with inactivating mutations in PMS2 or EXO1 have significant impairments in CSR because of defects in DSB formation in S regions ^75, 76^. PMS2- and EXO1-mediated excision of dU:dG mismatched DNA creates a DSB with a 5′ overhang that can be resolved into a blunt (or nearly blunt) DSB by DNA polymerases (η and θ), which subsequently is used by proteins of the C-NHEJ and A-EJ pathways to complete CSR ^74, 77, 78^.

### Positive feedback loop to amplify DNA breaks through AID phosphorylation

Despite the overwhelming genetic and biochemical data demonstrating the role of BER and MMR in CSR, the mechanism by which BER and MMR are subverted (or coopted) to promote recombinogenic repair of S regions rather than canonical repair remains uncharacterized. Hypothetically, AID may generate a high density of dU:dG mismatches within the S regions that cannot be repaired by canonical BER and MMR pathways. To maintain genomic integrity, BER and MMR are shunted toward recombinogenic repair and thus CSR. AID phosphorylation regulates the balance of canonical and recombinogenic repair that is mediated by BER and MMR downstream of AID-dependent deamination of S regions ^62^.

**Figure 3.**
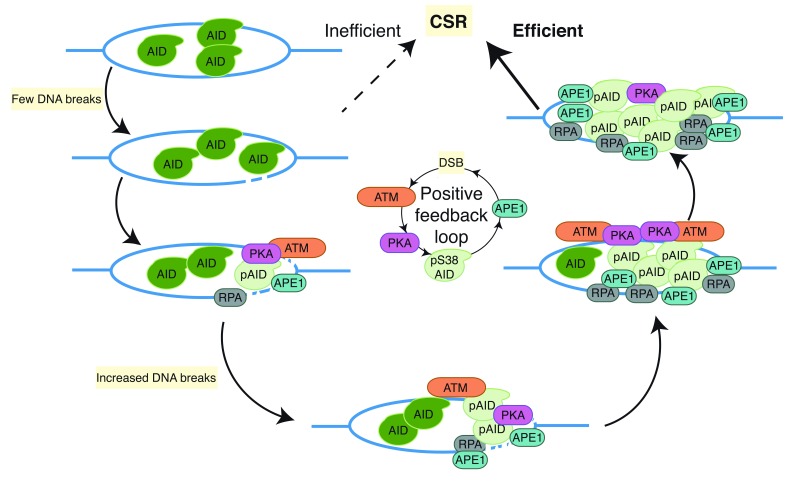
A hypothetical positive feedback loop generates a high density of DNA double-strand breaks to promote wild-type CSR. AID-mediated deamination of S regions generates DNA breaks that induce PKA-dependent AID phosphorylation at serine-38 (pS38-AID) and subsequent binding of APE1 and RPA to pS38-AID. Recruitment of APE1 to S regions generates additional DNA breaks, inducing additional AID phosphorylation through an unidentified ATM-dependent mechanism of activating PKA. AID, activation-induced cytidine daminase; APE1, apurinic/apyrimidinic endonuclease 1; ATM, ataxia telangiectasia mutated; CSR, class switch recombination; PKA, protein kinase A; RPA, replication protein A.

Phosphorylation of AID at Ser38 (pS38-AID) is critical for CSR as mice harboring a homozygous S38A knock-in mutation ( *AID ^S38A/S38A^*) have a significant reduction in CSR ^79, 80^. S38 lies within a consensus cAMP-dependent PKA phosphorylation site ^80– 82^. A hypomorphic PKA-RIα knock-in mutant ( *RIαB*) substantially impairs CSR and blocks phosphorylation of AID at S regions ^83^, indicating that PKA is required for AID phosphorylation at S38. Multiple isoforms of protein kinase C (PKC) can phosphorylate AID at S38 *in vitro* ^80^; however, the regulation of PKC-mediated AID phosphorylation *in vivo* remains unknown. Although the mutant AID (S38A) protein retains wild-type levels of deaminase activity *in vitro* and binding to S-region DNA *in vivo*, *AID ^S38A/S38A^* B cells cannot efficiently generate DSBs at recombining S regions ^62^. These data in conjunction with biochemical data demonstrating the indirect interaction of pS38-AID with APE1 strongly suggest that pS38-AID is required for DSB formation ^62^. Endogenous wild-type AID in *UNG ^−/−^MSH2 ^−/−^* B cells or catalytically inactive AID cannot be phosphorylated at S38 and consequently cannot bind to APE1; however, treating these cells with ionizing irradiation to induce DSBs restores both AID phosphorylation and APE1 binding, suggesting that the conversion of AID-dependent S-region DNA deamination into single-strand breaks by BER (APE1) or MMR (PMS2/EXO1) is required for AID phosphorylation. Thus, AID phosphorylation at S38 is required for, and dependent on, DNA breaks ^62^. These findings suggest the existence of a positive feedback loop wherein a low density of DNA breaks leads to AID phosphorylation, APE1 binding, and additional DNA breaks, which in turn activate more AID phosphorylation ( Figure 3). Consistent with this model, ATM, a serine/threonine protein kinase that activates DNA repair pathways in response to DSBs, is required for wild-type levels of AID phosphorylation and APE1 interaction ^62^. This model uncovers a previously undescribed role for ATM as a molecular rheostat that couples targeted DNA double-strand break formation with non-canonical, recombinogenic DNA repair to promote Ig gene diversification ( Figure 3).

## Resolution of DSBs

CSR requires joining DSBs in donor and acceptor S regions that may be separated by over 100 kb; however, some DSBs within an S region may be joined to another DSB within the same S region, resulting in an internal deletion rather than productive CSR ^45, 84, 85^. In addition, DSBs in S regions can be ligated to a DSB on another chromosome to generate a chromosomal translocation ^45^. The molecular mechanisms that promote the end joining of DSBs in distal S regions rather than canonical DNA repair, internal deletions, or chromosomal translocations remain largely unknown. During CSR, the *Igh* locus is re-organized into transcriptionally active loops, wherein I-promoters and regulatory enhancers (Eμ and Eα) are positioned close to one another to promote transcription, accessibility, and synapsis of recombining S regions ^84– 88^. Productive CSR is observed in B cells that have S regions replaced by I-SceI restriction sites ^89^, suggesting that the general cellular DNA damage repair pathways, which function in the synapsis and long-range end-joining of S-region DSBs, are required for the resolution of DSBs in S regions and productive CSR ^89^. Following the introduction of DSBs in the S regions, ATM and its substrate 53BP1 are thought to promote S-S region synapsis and recombinogenic repair ^60, 90, 91^. In the absence of ATM, DSBs at IgH and chromosomal translocations involving IgH are increased and CSR is decreased ^90– 92^. Recently, 53BP1 was shown to facilitate S-S synapsis, as Eα interactions with Eμ and the γ1 promoter are reduced in *53BP1 ^−/−^* B cells that are stimulated with LPS or LPS+IL4 ^60^.

### ATM-dependent and -independent DNA damage responses during CSR

**Figure 4.**
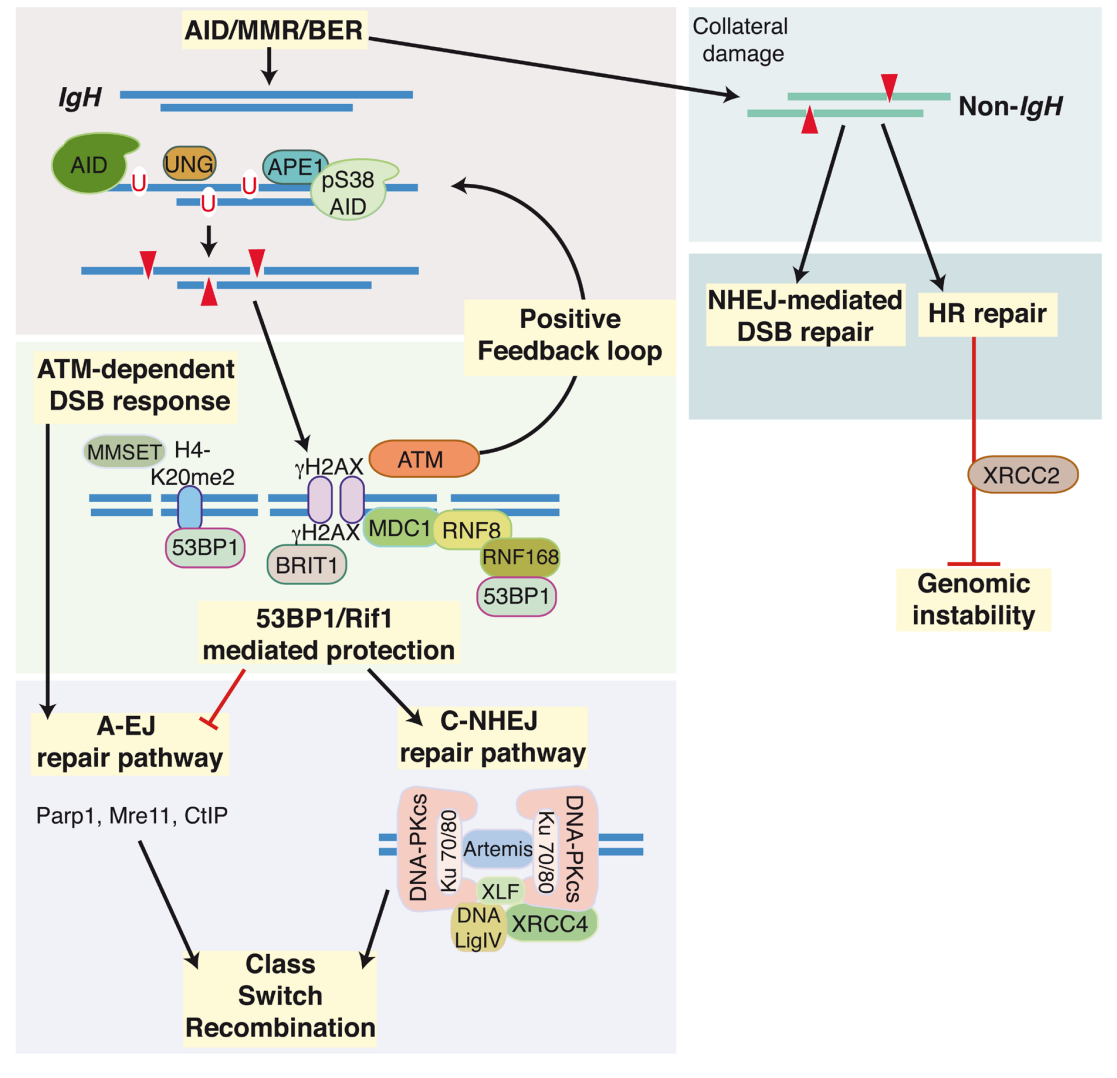
Resolution of DSBs generated by MMR or BER following AID-dependent deamination of S regions is accomplished by multiple pathways. ATM directly or indirectly phosphorylates proteins (for example, H2AX, MDC1, 53BP1, and AID) and stabilizes protein complexes that aid in the formation and resolution of DSBs during class switch recombination. A-EJ, alternative end-joining; AID, activation-induced cytidine daminase; BER, base excision repair; cNHEJ, classical non-homologous end-joining; DSB, double-strand break; HR, homologous recombination; MMR, mismatch repair.

ATM plays a role not only in the stabilization of S-region DSBs through the proposed synapsis and joining of S regions but also in the generation of DSBs through the phosphorylation of AID and the subsequent interaction of AID with APE1 ^62, 90– 92^. Activation of ATM kinase activity requires binding of the Mre11/RAD50/Nbs1 (MRN) complex to DSBs, which induces ATM-dependent phosphorylation of proteins mediating cell cycle checkpoints (for example, p53) and DNA repair, such as H2AX, MDC1, Nbs1, and 53BP1 ^60, 93, 94^. The DNA damage response initiated by ATM promotes the assembly of macromolecular foci flanking DSBs and provides docking sites for DNA repair proteins to bind and stabilize DNA ends to promote recombinogenic repair during CSR. Null mutations in ATM substrates impair CSR and increase chromosomal abnormalities and translocations ^90– 92^. 53BP1 deficiency leads to the most robust defect in CSR ^95– 97^. Mutation or deletion of 53BP1 results in a 90% defect in CSR with a significant proportion of chromosomal aberrations involving the *Igh* locus ^92, 98^ as well as a high frequency of Sμ internal deletions in cells stimulated for CSR ^99^. 53BP1 promotes the synapsis and long-range joining of S regions ^60^ and protects DNA ends from end resection to direct DNA repair toward NHEJ ^100, 101^. Consistent with these roles for 53BP1, ATM-mediated phosphorylation of 53BP1 recruits Rap-1 interacting factor (Rif1) to sites of DNA damage to protect DNA ends from resection and to promote DNA repair ^102^. Accordingly, Rif1-deficient B cells are significantly impaired in CSR ^102^ ( Figure 4). Additionally, 53BP1 can be recruited to S-region DSBs through ATM-independent pathways. 53BP1 interacts with H4K20me2 at sites of DNA damage. Depletion of the histone methyltransferase MMSET in the CH12F3 B cell line decreases both H4K20me2 levels and 53BP1 accumulation at S regions, thereby impairing CSR ^103, 104^. Furthermore, the recruitment of 53BP1 to DSBs has been shown to require the RNF8- and RNF168-dependent histone ubiquitination pathway. *RNF8 ^−/−^* and *RNF168 ^−/−^* B cells have decreased 53BP1 at S regions and a concomitant reduction in CSR ^105– 108^. Because CSR is more dramatically reduced in 53BP1-deficient B cells as compared to ATM-, H2AX-, MDC1-, or RNF8-deficient B cells, 53BP1 also has a function during CSR that is independent of the ATM/γH2AX/MDC1/RNF8 DNA damage response ^90, 91, 96, 97, 105– 110^. Data showing reduced Eα interactions with Eμ and the γ1 promoter in LPS- or LPS+IL4-stimulated *53BP1 ^−/−^* B cells demonstrate a role for 53BP1 in S-S synapsis during CSR ^60^.

More recently, BRCT-repeat inhibitor of hTert expression (BRIT1) has been implicated as a novel effector of the DNA repair phase of CSR ^111^. BRIT1 is a ubiquitously expressed protein that is rapidly recruited to DSBs after ionizing radiation through its C-terminal BRCT repeat domain, which is necessary for its interaction with phosphorylated H2AX (γH2AX) ^112^. As predicted, successful CSR requires BRIT1 interaction with γH2AX at recombining S regions ^111^. In addition, the BRIT1-γH2AX pathway is further modulated by the interaction of γH2AX with MDC1 in CSR. Although BRIT1 or MDC1 deficiency alone leads to a moderate reduction in CSR, loss of both BRIT1 and MDC1 together markedly impairs CSR ^111^. Thus, BRIT1 likely serves as a scaffold to recruit factors that resolve DSBs at S regions downstream of ATM ( Figure 4).

### End-joining

Homologous recombination (HR) and NHEJ are the two major pathways for DSB repair in mammalian cells ^113^. HR is restricted to the S/G _2_ phase of the cell cycle and requires large stretches of homology, whereas NHEJ is active throughout the cell cycle and requires little or no homology. Since CSR-associated DSBs are observed primarily during the G _1_ phase of the cell cycle and do not have extended stretches of homology, NHEJ is generally considered the major pathway in the joining of DSBs during CSR ^95, 113^. Consistent with this, mutations in the canonical NHEJ components Ku70/Ku80 heterodimer (Ku), XRCC4, and DNA ligase IV (Lig4) severely compromise CSR, while mutations in non-canonical NHEJ proteins such as DNA-dependent protein kinase catalytic subunit (DNA-PKcs), Artemis, and XRCC4-like factor (XLF or Cernunnos) increase chromosomal translocations even though CSR is not severely impaired ^95^.

B cells lacking core NHEJ components are capable of residual CSR, mediated by microhomology-biased A-EJ pathway ( Figure 4). Although A-EJ is a poorly defined DNA repair mechanism guided by microhomology between two DSBs, factors from other DNA repair pathways, including XRCC1, Ligase III, Mre11, Parp1, and CtIP, have been shown to be necessary for A-EJ during CSR ^114– 117^. More recently, Rad52, an HR repair factor, was shown to facilitate microhomology-mediated A-EJ that favors intra–S region recombination and competes with Ku to mediate inter–S region DSB recombination ^118^. Whether A-EJ is physiologically necessary in order to complete a productive class switch reaction is extensively discussed in 95.

While C-NHEJ and A-EJ are the primary end-joining pathways ligating DSBs within IgH during CSR, HR has been proposed to repair AID-induced off-target DSBs. Deficiency of the Rad51 paralog XRCC2, a key component of HR-mediated repair, significantly enhances AID-dependent genome-wide DNA damage ^119, 120^. Notably, AID-expressing human chronic lymphocytic leukemia cells are hypersensitive to HR inhibitors and this is possibly due to AID-dependent synthetic cytotoxicity from unrepaired DSBs at non-Ig loci ^121^. Thus, HR is essential for repairing AID-generated DSBs and dysregulated AID activity may provide a novel therapeutic approach to treat B cell malignancies.

## Concluding remarks

The discovery of AID as a master regulator of CSR and SHM revolutionized our understanding of Ig gene diversification and the mechanisms regulating genome integrity. The physiological targets of AID during CSR and SHM are almost exclusively restricted to S and V regions of the Ig loci, but AID can deaminate *in vitro* any transcribed substrate and damage *in vivo* many non-Ig genes, threatening genomic stability in B cells. However, B cells have evolved mechanisms that promote AID-dependent mutagenic and recombinogenic DNA repair within Ig loci while faithfully repairing collateral damage at non-Ig loci using canonical, conserved DNA repair pathways. Unlike V(D)J recombination, CSR has coopted the general DNA damage response to simultaneously generate and resolve DSBs within S regions. BER and MMR are essential, complementary pathways for CSR. ATM functions as a generator of DSBs in S regions, an essential signaling molecule to mobilize DNA repair proteins, and a scaffold for these proteins to resolve DSBs. As additional CSR factors, such as RNF8/168 and BRIT1, are identified, we will further understand the genetic and molecular mechanisms regulating the formation and repair of DSBs during CSR.

## References

[1] Jung D Alt FW: Unraveling V(D)J recombination; insights into gene regulation. *Cell.* 2004;116(2):299–311. 10.1016/S0092-8674(04)00039-X 14744439

[2] Schatz DG Ji Y: Recombination centres and the orchestration of V(D)J recombination. *Nat Rev Immunol.* 2011;11(4):251–63. 10.1038/nri2941 21394103

[3] Methot SP Di Noia JM: Molecular Mechanisms of Somatic Hypermutation and Class Switch Recombination. *Adv Immunol.* 2017;133:37–87. 10.1016/bs.ai.2016.11.002 28215280

[4] Hwang JK Alt FW Yeap LS: Related Mechanisms of Antibody Somatic Hypermutation and Class Switch Recombination. *Microbiol Spectr.* 2015;3(1):MDNA3-0037-2014. 10.1128/microbiolspec.MDNA3-0037-2014 26104555 PMC4481323

[5] Choi JH Wang KW Zhang D: IgD class switching is initiated by microbiota and limited to mucosa-associated lymphoid tissue in mice. *Proc Natl Acad Sci U S A.* 2017;114(7):E1196–E1204. 10.1073/pnas.1621258114 28137874 PMC5321007

[6] Zanotti KJ Gearhart PJ: Antibody diversification caused by disrupted mismatch repair and promiscuous DNA polymerases. *DNA Repair (Amst).* 2016;38:110–6. 10.1016/j.dnarep.2015.11.011 26719140 PMC4740194

[7] Muramatsu M Kinoshita K Fagarasan S: Class switch recombination and hypermutation require activation-induced cytidine deaminase (AID), a potential RNA editing enzyme. *Cell.* 2000;102(5):553–63. 10.1016/S0092-8674(00)00078-7 11007474

[8] Muramatsu M Sankaranand VS Anant S: Specific expression of activation-induced cytidine deaminase (AID), a novel member of the RNA-editing deaminase family in germinal center B cells. *J Biol Chem.* 1999;274(26):18470–6. 10.1074/jbc.274.26.18470 10373455

[9] Rada C Di Noia JM Neuberger MS: Mismatch recognition and uracil excision provide complementary paths to both Ig switching and the A/T-focused phase of somatic mutation. *Mol Cell.* 2004;16(2):163–71. 10.1016/j.molcel.2004.10.011 15494304

[10] Liu M Duke JL Richter DJ: Two levels of protection for the B cell genome during somatic hypermutation. *Nature.* 2008;451(7180):841–5. 10.1038/nature06547 18273020

[11] Pasqualucci L Neumeister P Goossens T: Hypermutation of multiple proto-oncogenes in B-cell diffuse large-cell lymphomas. *Nature.* 2001;412(6844):341–6. 10.1038/35085588 11460166

[12] Robbiani DF Bothmer A Callen E: AID is required for the chromosomal breaks in *c-myc* that lead to *c-myc/IgH* translocations. *Cell.* 2008;135(6):1028–38. 10.1016/j.cell.2008.09.062 19070574 PMC2713603

[13] Shen HM Peters A Baron B: Mutation of *BCL-6* gene in normal B cells by the process of somatic hypermutation of Ig genes. *Science.* 1998;280(5370):1750–2. 10.1126/science.280.5370.1750 9624052

[14] Yamane A Resch W Kuo N: Deep-sequencing identification of the genomic targets of the cytidine deaminase AID and its cofactor RPA in B lymphocytes. *Nat Immunol.* 2011;12(1):62–9. 10.1038/ni.1964 21113164 PMC3005028

[15] Nussenzweig A Nussenzweig MC: Origin of chromosomal translocations in lymphoid cancer. *Cell.* 2010;141(1):27–38. 10.1016/j.cell.2010.03.016 20371343 PMC2874895

[16] Pasqualucci L Bhagat G Jankovic M: AID is required for germinal center-derived lymphomagenesis. *Nat Genet.* 2008;40(1):108–12. 10.1038/ng.2007.35 18066064

[17] Rocha PP Skok JA: The origin of recurrent translocations in recombining lymphocytes: a balance between break frequency and nuclear proximity. *Curr Opin Cell Biol.* 2013;25(3):365–71. 10.1016/j.ceb.2013.02.007 23478218 PMC3691303

[18] Chaudhuri J Alt FW: Class-switch recombination: interplay of transcription, DNA deamination and DNA repair. *Nat Rev Immunol.* 2004;4(7):541–52. 10.1038/nri1395 15229473

[19] Chaudhuri J Basu U Zarrin A: Evolution of the immunoglobulin heavy chain class switch recombination mechanism. *Adv Immunol.* 2007;94:157–214. 10.1016/S0065-2776(06)94006-1 17560275

[20] Jung S Rajewsky K Radbruch A: Shutdown of class switch recombination by deletion of a switch region control element. *Science.* 1993;259(5097):984–7. 10.1126/science.8438159 8438159

[21] Zhang J Bottaro A Li S: A selective defect in IgG2b switching as a result of targeted mutation of the I gamma 2b promoter and exon. *EMBO J.* 1993;12(9):3529–37. 8253079 10.1002/j.1460-2075.1993.tb06027.x PMC413629

[22] Khamlichi AA Glaudet F Oruc Z: Immunoglobulin class-switch recombination in mice devoid of any S mu tandem repeat. *Blood.* 2004;103(10):3828–36. 10.1182/blood-2003-10-3470 14962903

[23] Luby TM Schrader CE Stavnezer J: The mu switch region tandem repeats are important, but not required, for antibody class switch recombination. *J Exp Med.* 2001;193(2):159–68. 10.1084/jem.193.2.159 11148220 PMC2193334

[24] Shinkura R Tian M Smith M: The influence of transcriptional orientation on endogenous switch region function. *Nat Immunol.* 2003;4(5):435–41. 10.1038/ni918 12679811

[25] Zarrin AA Tian M Wang J: Influence of switch region length on immunoglobulin class switch recombination. *Proc Natl Acad Sci U S A.* 2005;102(7):2466–70. 10.1073/pnas.0409847102 15684074 PMC548964

[26] Qiao Q Wang L Meng FL: AID Recognizes Structured DNA for Class Switch Recombination. *Mol Cell.* 2017;67(3):361–373.e4. 10.1016/j.molcel.2017.06.034 28757211 PMC5771415

[27] Xu Z Fulop Z Wu G: 14-3-3 adaptor proteins recruit AID to 5'-AGCT-3'-rich switch regions for class switch recombination. *Nat Struct Mol Biol.* 2010;17(9):1124–35. 10.1038/nsmb.1884 20729863 PMC3645988

[28] Xu Z Zan H Pone EJ: Immunoglobulin class-switch DNA recombination: induction, targeting and beyond. *Nat Rev Immunol.* 2012;12(7):517–31. 10.1038/nri3216 22728528 PMC3545482

[29] Bransteitter R Pham P Scharff MD: Activation-induced cytidine deaminase deaminates deoxycytidine on single-stranded DNA but requires the action of RNase. *Proc Natl Acad Sci U S A.* 2003;100(7):4102–7. 10.1073/pnas.0730835100 12651944 PMC153055

[30] Chaudhuri J Tian M Khuong C: Transcription-targeted DNA deamination by the AID antibody diversification enzyme. *Nature.* 2003;422(6933):726–30. 10.1038/nature01574 12692563

[31] Ramiro AR Stavropoulos P Jankovic M: Transcription enhances AID-mediated cytidine deamination by exposing single-stranded DNA on the nontemplate strand. *Nat Immunol.* 2003;4(5):452–6. 10.1038/ni920 12692548

[32] Yu K Chedin F Hsieh CL: R-loops at immunoglobulin class switch regions in the chromosomes of stimulated B cells. *Nat Immunol.* 2003;4(5):442–51. 10.1038/ni919 12679812

[33] Zheng S Vuong BQ Vaidyanathan B: Non-coding RNA Generated following Lariat Debranching Mediates Targeting of AID to DNA. *Cell.* 2015;161(4):762–73. 10.1016/j.cell.2015.03.020 25957684 PMC4426339

[34] Yang Y McBride KM Hensley S: Arginine methylation facilitates the recruitment of TOP3B to chromatin to prevent R loop accumulation. *Mol Cell.* 2014;53(3):484–97. 10.1016/j.molcel.2014.01.011 24507716 PMC3959860

[35] Pefanis E Wang J Rothschild G: Noncoding RNA transcription targets AID to divergently transcribed loci in B cells. *Nature.* 2014;514(7522):389–93. 10.1038/nature13580 25119026 PMC4372240

[36] Pavri R Gazumyan A Jankovic M: Activation-induced cytidine deaminase targets DNA at sites of RNA polymerase II stalling by interaction with Spt5. *Cell.* 2010;143(1):122–33. 10.1016/j.cell.2010.09.017 20887897 PMC2993080

[37] Willmann KL Milosevic S Pauklin S: A role for the RNA pol II-associated PAF complex in AID-induced immune diversification. *J Exp Med.* 2012;209(11):2099–111. 10.1084/jem.20112145 23008333 PMC3478926

[38] Aida M Hamad N Stanlie A: Accumulation of the FACT complex, as well as histone H3.3, serves as a target marker for somatic hypermutation. *Proc Natl Acad Sci U S A.* 2013;110(19):7784–9. 10.1073/pnas.1305859110 23610419 PMC3651492

[39] Basu U Meng FL Keim C: The RNA exosome targets the AID cytidine deaminase to both strands of transcribed duplex DNA substrates. *Cell.* 2011;144(3):353–63. 10.1016/j.cell.2011.01.001 21255825 PMC3065114

[40] Wang X Fan M Kalis S: A source of the single-stranded DNA substrate for activation-induced deaminase during somatic hypermutation. *Nat Commun.* 2014;5: 4137. 10.1038/ncomms5137 24923561 PMC4154566

[41] Hein K Lorenz MG Siebenkotten G: Processing of switch transcripts is required for targeting of antibody class switch recombination. *J Exp Med.* 1998;188(12):2369–74. 10.1084/jem.188.12.2369 9858523 PMC2212419

[42] Lorenz M Jung S Radbruch A: Switch transcripts in immunoglobulin class switching. *Science.* 1995;267(5205):1825–8. 10.1126/science.7892607 7892607

[43] Lorenz MG Radbruch A: Insights into the control of immunoglobulin class switch recombination from analysis of targeted mice. *Res Immunol.* 1997;148(7):460–3. 10.1016/S0923-2494(97)82671-5 9498006

[44] Conticello SG Ganesh K Xue K: Interaction between antibody-diversification enzyme AID and spliceosome-associated factor CTNNBL1. *Mol Cell.* 2008;31(4):474–84. 10.1016/j.molcel.2008.07.009 18722174

[45] Matthews AJ Zheng S DiMenna LJ: Regulation of immunoglobulin class-switch recombination: choreography of noncoding transcription, targeted DNA deamination, and long-range DNA repair. *Adv Immunol.* 2014;122:1–57. 10.1016/B978-0-12-800267-4.00001-8 24507154 PMC4150736

[46] Nowak U Matthews AJ Zheng S: The splicing regulator PTBP2 interacts with the cytidine deaminase AID and promotes binding of AID to switch-region DNA. *Nat Immunol.* 2011;12(2):160–6. 10.1038/ni.1977 21186367 PMC3724472

[47] Müller JR Marcu KB: Stimulation of murine B lymphocytes induces a DNA exonuclease whose activity on switch-mu DNA is specifically inhibited by other germ-line switch region RNAs. *J Immunol.* 1998;160(7):3337–41. 9531292

[48] Yewdell WT Chaudhuri J: A transcriptional serenAID: the role of noncoding RNAs in class switch recombination. *Int Immunol.* 2017;29(4):183–96. 10.1093/intimm/dxx027 28535205 PMC5890902

[49] Ruskin B Green MR: An RNA processing activity that debranches RNA lariats. *Science.* 1985;229(4709):135–40. 10.1126/science.2990042 2990042

[50] DiMenna LJ Chaudhuri J: Regulating infidelity: RNA-mediated recruitment of AID to DNA during class switch recombination. *Eur J Immunol.* 2016;46(3):523–30. 10.1002/eji.201545809 26799454 PMC5373104

[51] Mahdaviani SA Hirbod-Mobarakeh A Wang N: Novel mutation of the activation-induced cytidine deaminase gene in a Tajik family: special review on hyper-immunoglobulin M syndrome. *Expert Rev Clin Immunol.* 2012;8(6):539–46. 10.1586/eci.12.46 22992148

[52] Li G Zan H Xu Z: Epigenetics of the antibody response. *Trends Immunol.* 2013;34(9):460–70. 10.1016/j.it.2013.03.006 23643790 PMC3744588

[53] Schotta G Sengupta R Kubicek S: A chromatin-wide transition to H4K20 monomethylation impairs genome integrity and programmed DNA rearrangements in the mouse. *Genes Dev.* 2008;22(15):2048–61. 10.1101/gad.476008 18676810 PMC2492754

[54] Rodríguez-Cortez VC Martínez-Redondo P Català-Moll F: Activation-induced cytidine deaminase targets SUV4-20-mediated histone H4K20 trimethylation to class-switch recombination sites. *Sci Rep.* 2017;7(1):7594. 10.1038/s41598-017-07380-9 28790320 PMC5548798

[55] Jeevan-Raj BP Robert I Heyer V: Epigenetic tethering of AID to the donor switch region during immunoglobulin class switch recombination. *J Exp Med.* 2011;208(8):1649–60. 10.1084/jem.20110118 21746811 PMC3149220

[56] Li G White CA Lam T: Combinatorial H3K9acS10ph histone modification in *IgH* locus S regions targets 14-3-3 adaptors and AID to specify antibody class-switch DNA recombination. *Cell Rep.* 2013;5(3):702–14. 10.1016/j.celrep.2013.09.031 24209747 PMC3951903

[57] Daniel JA Santos MA Wang Z: PTIP promotes chromatin changes critical for immunoglobulin class switch recombination. *Science.* 2010;329(5994):917–23. 10.1126/science.1187942 20671152 PMC3008398

[58] Kuang FL Luo Z Scharff MD: H3 trimethyl K9 and H3 acetyl K9 chromatin modifications are associated with class switch recombination. *Proc Natl Acad Sci U S A.* 2009;106(13):5288–93. 10.1073/pnas.0901368106 19276123 PMC2654022

[59] Wang L Wuerffel R Feldman S: S region sequence, RNA polymerase II, and histone modifications create chromatin accessibility during class switch recombination. *J Exp Med.* 2009;206(8):1817–30. 10.1084/jem.20081678 19596805 PMC2722165

[60] Feldman S Wuerffel R Achour I: 53BP1 Contributes to *Igh* Locus Chromatin Topology during Class Switch Recombination. *J Immunol.* 2017;198(6):2434–44. 10.4049/jimmunol.1601947 28159901 PMC5695034

[61] Bonaud A Lechouane F Le Noir S: Efficient AID targeting of switch regions is not sufficient for optimal class switch recombination. *Nat Commun.* 2015;6: 7613. 10.1038/ncomms8613 26146363

[62] Vuong BQ Herrick-Reynolds K Vaidyanathan B: A DNA break- and phosphorylation-dependent positive feedback loop promotes immunoglobulin class-switch recombination. *Nat Immunol.* 2013;14(11):1183–9. 10.1038/ni.2732 24097111 PMC4005274

[63] Di Noia JM Williams GT Chan DT: Dependence of antibody gene diversification on uracil excision. *J Exp Med.* 2007;204(13):3209–19. 10.1084/jem.20071768 18070939 PMC2150978

[64] Guikema JE Linehan EK Tsuchimoto D: APE1- and APE2-dependent DNA breaks in immunoglobulin class switch recombination. *J Exp Med.* 2007;204(12):3017–26. 10.1084/jem.20071289 18025127 PMC2118529

[65] Petersen-Mahrt SK Harris RS Neuberger MS: AID mutates *E. coli* suggesting a DNA deamination mechanism for antibody diversification. *Nature.* 2002;418(6893):99–103. 10.1038/nature00862 12097915

[66] Rada C Williams GT Nilsen H: Immunoglobulin isotype switching is inhibited and somatic hypermutation perturbed in UNG-deficient mice. *Curr Biol.* 2002;12(20):1748–55. 10.1016/S0960-9822(02)01215-0 12401169

[67] Di Noia J Neuberger MS: Altering the pathway of immunoglobulin hypermutation by inhibiting uracil-DNA glycosylase. *Nature.* 2002;419(6902):43–8. 10.1038/nature00981 12214226

[68] Imai K Slupphaug G Lee WI: Human uracil-DNA glycosylase deficiency associated with profoundly impaired immunoglobulin class-switch recombination. *Nat Immunol.* 2003;4(10):1023–8. 10.1038/ni974 12958596

[69] Schrader CE Linehan EK Mochegova SN: Inducible DNA breaks in Ig S regions are dependent on AID and UNG. *J Exp Med.* 2005;202(4):561–8. 10.1084/jem.20050872 16103411 PMC2212854

[70] Zan H White CA Thomas LM: Rev1 recruits ung to switch regions and enhances du glycosylation for immunoglobulin class switch DNA recombination. *Cell Rep.* 2012;2(5):1220–32. 10.1016/j.celrep.2012.09.029 23140944 PMC3518390

[71] Guikema JE Stavnezer J Schrader CE: The role of Apex2 in class-switch recombination of immunoglobulin genes. *Int Immunol.* 2010;22(3):213; author reply 213–4. 10.1093/intimm/dxq003 20185435

[72] Masani S Han L Yu K: Apurinic/apyrimidinic endonuclease 1 is the essential nuclease during immunoglobulin class switch recombination. *Mol Cell Biol.* 2013;33(7):1468–73. 10.1128/MCB.00026-13 23382073 PMC3624277

[73] Schrader CE Guikema JE Wu X: The roles of APE1, APE2, DNA polymerase beta and mismatch repair in creating S region DNA breaks during antibody class switch. *Philos Trans R Soc Lond B Biol Sci.* 2009;364(1517):645–52. 10.1098/rstb.2008.0200 19010771 PMC2660920

[74] Stavnezer J Guikema JE Schrader CE: Mechanism and regulation of class switch recombination. *Annu Rev Immunol.* 2008;26:261–92. 10.1146/annurev.immunol.26.021607.090248 18370922 PMC2707252

[75] Bardwell PD Woo CJ Wei K: Altered somatic hypermutation and reduced class-switch recombination in exonuclease 1-mutant mice. *Nat Immunol.* 2004;5(2):224–9. 10.1038/ni1031 14716311

[76] Péron S Metin A Gardès P: Human PMS2 deficiency is associated with impaired immunoglobulin class switch recombination. *J Exp Med.* 2008;205(11):2465–72. 10.1084/jem.20080789 18824584 PMC2571921

[77] Eccleston J Yan C Yuan K: Mismatch repair proteins MSH2, MLH1, and EXO1 are important for class-switch recombination events occurring in B cells that lack nonhomologous end joining. *J Immunol.* 2011;186(4):2336–43. 10.4049/jimmunol.1003104 21242524 PMC3072809

[78] Min IM Schrader CE Vardo J: The Smu tandem repeat region is critical for Ig isotype switching in the absence of Msh2. *Immunity.* 2003;19(4):515–24. 10.1016/S1074-7613(03)00262-0 14563316

[79] Cheng HL Vuong BQ Basu U: Integrity of the AID serine-38 phosphorylation site is critical for class switch recombination and somatic hypermutation in mice. *Proc Natl Acad Sci U S A.* 2009;106(8):2717–22. 10.1073/pnas.0812304106 19196992 PMC2650332

[80] McBride KM Gazumyan A Woo EM: Regulation of class switch recombination and somatic mutation by AID phosphorylation. *J Exp Med.* 2008;205(11):2585–94. 10.1084/jem.20081319 18838546 PMC2571933

[81] Basu U Chaudhuri J Alpert C: The AID antibody diversification enzyme is regulated by protein kinase A phosphorylation. *Nature.* 2005;438(7067):508–11. 10.1038/nature04255 16251902

[82] Pasqualucci L Kitaura Y Gu H: PKA-mediated phosphorylation regulates the function of activation-induced deaminase (AID) in B cells. *Proc Natl Acad Sci U S A.* 2006;103(2):395–400. 10.1073/pnas.0509969103 16387847 PMC1326186

[83] Vuong BQ Lee M Kabir S: Specific recruitment of protein kinase A to the immunoglobulin locus regulates class-switch recombination. *Nat Immunol.* 2009;10(4):420–6. 10.1038/ni.1708 19234474 PMC4169875

[84] Alt FW Rosenberg N Casanova RJ: Immunoglobulin heavy-chain expression and class switching in a murine leukaemia cell line. *Nature.* 1982;296(5855):325–31. 10.1038/296325a0 6801527

[85] Gu H Zou YR Rajewsky K: Independent control of immunoglobulin switch recombination at individual switch regions evidenced through Cre- *loxP*-mediated gene targeting. *Cell.* 1993;73(6):1155–64. 10.1016/0092-8674(93)90644-6 8513499

[86] Ju Z Volpi SA Hassan R: Evidence for physical interaction between the immunoglobulin heavy chain variable region and the 3' regulatory region. *J Biol Chem.* 2007;282(48):35169–78. 10.1074/jbc.M705719200 17921139

[87] Kenter AL Feldman S Wuerffel R: Three-dimensional architecture of the IgH locus facilitates class switch recombination. *Ann N Y Acad Sci.* 2012;1267(1):86–94. 10.1111/j.1749-6632.2012.06604.x 22954221 PMC3442954

[88] Wuerffel R Wang L Grigera F: S-S synapsis during class switch recombination is promoted by distantly located transcriptional elements and activation-induced deaminase. *Immunity.* 2007;27(5):711–22. 10.1016/j.immuni.2007.09.007 17980632 PMC4979535

[89] Zarrin AA Del Vecchio C Tseng E: Antibody class switching mediated by yeast endonuclease-generated DNA breaks. *Science.* 2007;315(5810):377–81. 10.1126/science.1136386 17170253

[90] Lumsden JM McCarty T Petiniot LK: Immunoglobulin class switch recombination is impaired in *Atm*-deficient mice. *J Exp Med.* 2004;200(9):1111–21. 10.1084/jem.20041074 15504820 PMC2211853

[91] Reina-San-Martin B Chen HT Nussenzweig A: ATM is required for efficient recombination between immunoglobulin switch regions. *J Exp Med.* 2004;200(9):1103–10. 10.1084/jem.20041162 15520243 PMC2211855

[92] Franco S Gostissa M Zha S: H2AX prevents DNA breaks from progressing to chromosome breaks and translocations. *Mol Cell.* 2006;21(2):201–14. 10.1016/j.molcel.2006.01.005 16427010

[93] Blackford AN Jackson SP: ATM, ATR, and DNA-PK: The Trinity at the Heart of the DNA Damage Response. *Mol Cell.* 2017;66(6):801–17. 10.1016/j.molcel.2017.05.015 28622525

[94] Paull TT: Mechanisms of ATM Activation. *Annu Rev Biochem.* 2015;84:711–38. 10.1146/annurev-biochem-060614-034335 25580527

[95] Boboila C Alt FW Schwer B: Classical and alternative end-joining pathways for repair of lymphocyte-specific and general DNA double-strand breaks. *Adv Immunol.* 2012;116:1–49. 10.1016/B978-0-12-394300-2.00001-6 23063072

[96] Manis JP Morales JC Xia Z: 53BP1 links DNA damage-response pathways to immunoglobulin heavy chain class-switch recombination. *Nat Immunol.* 2004;5(5):481–7. 10.1038/ni1067 15077110

[97] Ward IM Reina-San-Martin B Olaru A: 53BP1 is required for class switch recombination. *J Cell Biol.* 2004;165(4):459–64. 10.1083/jcb.200403021 15159415 PMC2172356

[98] Adams MM Carpenter PB: Tying the loose ends together in DNA double strand break repair with 53BP1. *Cell Div.* 2006;1:19. 10.1186/1747-1028-1-19 16945145 PMC1601952

[99] Reina-San-Martin B Chen J Nussenzweig A: Enhanced intra-switch region recombination during immunoglobulin class switch recombination in 53BP1 ^-/-^ B cells. *Eur J Immunol.* 2007;37(1):235–9. 10.1002/eji.200636789 17183606

[100] Bothmer A Robbiani DF Di Virgilio M: Regulation of DNA end joining, resection, and immunoglobulin class switch recombination by 53BP1. *Mol Cell.* 2011;42(3):319–29. 10.1016/j.molcel.2011.03.019 21549309 PMC3142663

[101] Bothmer A Robbiani DF Feldhahn N: 53BP1 regulates DNA resection and the choice between classical and alternative end joining during class switch recombination. *J Exp Med.* 2010;207(4):855–65. 10.1084/jem.20100244 20368578 PMC2856023

[102] Di Virgilio M Callen E Yamane A: Rif1 prevents resection of DNA breaks and promotes immunoglobulin class switching. *Science.* 2013;339(6120):711–5. 10.1126/science.1230624 23306439 PMC3815530

[103] Hajdu I Ciccia A Lewis SM: Wolf-Hirschhorn syndrome candidate 1 is involved in the cellular response to DNA damage. *Proc Natl Acad Sci U S A.* 2011;108(32):13130–4. 10.1073/pnas.1110081108 21788515 PMC3156169

[104] Pei H Zhang L Luo K: MMSET regulates histone H4K20 methylation and 53BP1 accumulation at DNA damage sites. *Nature.* 2011;470(7332):124–8. 10.1038/nature09658 21293379 PMC3064261

[105] Bohgaki T Bohgaki M Cardoso R: Genomic instability, defective spermatogenesis, immunodeficiency, and cancer in a mouse model of the RIDDLE syndrome. *PLoS Genet.* 2011;7(4):e1001381. 10.1371/journal.pgen.1001381 21552324 PMC3084200

[106] Li L Halaby M Hakem A: Rnf8 deficiency impairs class switch recombination, spermatogenesis, and genomic integrity and predisposes for cancer. *J Exp Med.* 2010;207(5):983–97. 10.1084/jem.20092437 20385750 PMC2867283

[107] Ramachandran S Chahwan R Nepal RM: The RNF8/RNF168 ubiquitin ligase cascade facilitates class switch recombination. *Proc Natl Acad Sci U S A.* 2010;107(2):809–14. 10.1073/pnas.0913790107 20080757 PMC2818930

[108] Santos MA Huen MS Jankovic M: Class switching and meiotic defects in mice lacking the E3 ubiquitin ligase RNF8. *J Exp Med.* 2010;207(5):973–81. 10.1084/jem.20092308 20385748 PMC2867275

[109] Lou Z Minter-Dykhouse K Franco S: MDC1 maintains genomic stability by participating in the amplification of ATM-dependent DNA damage signals. *Mol Cell.* 2006;21(2):187–200. 10.1016/j.molcel.2005.11.025 16427009

[110] Reina-San-Martin B Difilippantonio S Hanitsch L: H2AX is required for recombination between immunoglobulin switch regions but not for intra-switch region recombination or somatic hypermutation. *J Exp Med.* 2003;197(12):1767–78. 10.1084/jem.20030569 12810694 PMC2193951

[111] Yen WF Chaudhry A Vaidyanathan B: BRCT-domain protein BRIT1 influences class switch recombination. *Proc Natl Acad Sci U S A.* 2017;114(31):8354–8359, pii: 201708211. 10.1073/pnas.1708211114 28724724 PMC5547652

[112] Jeffers LJ Coull BJ Stack SJ: Distinct BRCT domains in Mcph1/Brit1 mediate ionizing radiation-induced focus formation and centrosomal localization. *Oncogene.* 2008;27(1):139–44. 10.1038/sj.onc.1210595 17599047

[113] Alt FW Zhang Y Meng FL: Mechanisms of programmed DNA lesions and genomic instability in the immune system. *Cell.* 2013;152(3):417–29. 10.1016/j.cell.2013.01.007 23374339 PMC4382911

[114] Dinkelmann M Spehalski E Stoneham T: Multiple functions of MRN in end-joining pathways during isotype class switching. *Nat Struct Mol Biol.* 2009;16(8):808–13. 10.1038/nsmb.1639 19633670 PMC2721910

[115] Lee-Theilen M Matthews AJ Kelly D: CtIP promotes microhomology-mediated alternative end joining during class-switch recombination. *Nat Struct Mol Biol.* 2011;18(1):75–9. 10.1038/nsmb.1942 21131982 PMC3471154

[116] Robert I Dantzer F Reina-San-Martin B: Parp1 facilitates alternative NHEJ, whereas Parp2 suppresses IgH/c-myc translocations during immunoglobulin class switch recombination. *J Exp Med.* 2009;206(5):1047–56. 10.1084/jem.20082468 19364882 PMC2715026

[117] Xie A Kwok A Scully R: Role of mammalian Mre11 in classical and alternative nonhomologous end joining. *Nat Struct Mol Biol.* 2009;16(8):814–8. 10.1038/nsmb.1640 19633669 PMC2730592

[118] Zan H Tat C Qiu Z: Rad52 competes with Ku70/Ku86 for binding to S-region DSB ends to modulate antibody class-switch DNA recombination. *Nat Commun.* 2017;8:14244. 10.1038/ncomms14244 28176781 PMC5309807

[119] Hasham MG Donghia NM Coffey E: Widespread genomic breaks generated by activation-induced cytidine deaminase are prevented by homologous recombination. *Nat Immunol.* 2010;11(9):820–6. 10.1038/ni.1909 20657597 PMC2930818

[120] Hasham MG Snow KJ Donghia NM: Activation-induced cytidine deaminase-initiated off-target DNA breaks are detected and resolved during S phase. *J Immunol.* 2012;189(5):2374–82. 10.4049/jimmunol.1200414 22826323 PMC3424338

[121] Lamont KR Hasham MG Donghia NM: Attenuating homologous recombination stimulates an AID-induced antileukemic effect. *J Exp Med.* 2013;210(5):1021–33. 10.1084/jem.20121258 23589568 PMC3646491

